# Editorial Note: Bioactive lipids in intervertebral disc (IVD) degeneration and its therapeutic implications

**DOI:** 10.1042/BSR-2019-2117_EDN

**Published:** 2024-08-28

**Keywords:** degeneration, eicosanoids, inflammation, intervertebral disc, lipoxin A4, prostaglandin E2

This commentary “Bioactive lipids in intervertebral disc degeneration and its therapeutic implications” was originally written for the now retracted article “High glucose promotes annulus fibrosus cell apoptosis through activating the JNK and p38 MAPK pathways” by Shan et al, 2019 (DOI: 10.1042/BSR20190853).

The Editorial Board feel that the commentary discusses the wider field and still provides a valuable contribution. Therefore, the commentary remains published despite the retraction of the original article.

